# HTLV-1-mediated dysregulation of the Wnt pathways: roles of Tax and HBZ

**DOI:** 10.1186/1742-4690-11-S1-P91

**Published:** 2014-01-07

**Authors:** Guangyong Ma, Jun-ichiro Yasunaga, Jun Fan, Shin-ichi Yanagawa, Masao Matsuoka

**Affiliations:** 1 Laboratory of Virus Control, Institute for Virus Research, Kyoto University, Kyoto, Japan; 2 Laboratory of Gene Analysis, Institute for Virus Research, Kyoto University, Kyoto, Japan

## 

Human T cell leukemia virus type 1 (HTLV-1) is the causative agent of adult T-cell leukemia (ATL). Dysregulations of cellular signaling pathways have been considered to be important strategies for HTLV-1 induced leukemogenesis. In this study, we found that a Wnt pathway-related protein, Dishevelled (Dvl)-associating protein with a high frequency of leucine residues (DAPLE), could be associated with Tax to activate the canonical Wnt signaling. However, in the downstream of the pathway, T-cell factor 1 (TCF1) and lymphoid enhancer-binding factor 1 (LEF1), which are two transcription factors mainly expressed in T-cells, could interact with Tax and suppress its transactivating ability. We further found virus production was greatly inhibited by TCF1/LEF1. Due to the high expression of TCF1/LEF1 in the thymus and their significant downregulation upon T-cell activation, we suspect TCF1/LEF1 may contribute to the HTLV-1 tropism on activated mature T-cells. On the other hand, HTLV-1 bZIP factor (HBZ) markedly suppressed canonical Wnt activation through targeting both TCF1 and LEF1. As a result, the canonical Wnt pathway was not activated in HTLV-1-infected cells, whereas the representative of noncanonical Wnt ligands, Wnt5a, which antagonizes canonical Wnt signaling, was overexpressed in HTLV-1 infected cells. Further experiments revealed HBZ could induce Wnt5a expression through activation of the TGF-β pathway. Importantly, knocking down of Wnt5a in HTLV-1-infected cells repressed cellular proliferation and migration. By suppressing the canonical and enhancing the noncanonical Wnt pathway, HBZ is likely able to generate a favorable environment for HTLV-1 infection and persistence.

